# Educational Programme on Knowledge, Attitudes, and Practice of Oral Care/Hygiene Provision by Healthcare Providers to Older Residents in Long-Term Care Institutions: A Case-Control Study

**DOI:** 10.3390/geriatrics9010016

**Published:** 2024-01-29

**Authors:** Florence M. F. Wong, Henry W. H. Shie, Enoch Kao, Hoi Mei Tsoi, Wai Keung Leung

**Affiliations:** 1 Tung Wah College, Hong Kong, China; 2 Hiu Kwong Groups Limited, Hong Kong, China; henry.shie@hiukwong.com; 3 Department of Microbiology, The University of Hong Kong, Hong Kong, China; enocow@connect.hku.hk; 4 Faculty of Dentistry, The University of Hong Kong, Hong Kong, China; u3556569@connect.hku.hk (H.M.T.); ewkleung@hku.hk (W.K.L.)

**Keywords:** oral care, older, knowledge, attitudes, practice, healthcare providers, long-term care institutions

## Abstract

Background: Much attention has been paid to advocate proper oral care/hygiene provision by healthcare providers in long-term care institutions (LTCIs). This study aimed to evaluate the effects of an oral health education (OHE) programme (intervention) on knowledge, attitudes, and practice (KAP) of healthcare providers in providing oral care/hygiene to older residents in LTCIs. Methods: A case control study was conducted at two LTCIs, with one assigned as the intervention group and the other as the control group. A KAP survey was administered before and after the intervention, and oral status was assessed by standardized clinical photographs taken before and after oral hygiene provision on three older residents. Results: A total of 40 healthcare providers (20 in intervention and 20 in control groups) participated, with the attitudes and overall KAP significantly improved in the intervention group after the OHE programme. Interestingly, the knowledge of those in the control LTCI was significantly declined at re-evaluation (mean scores were from 17.25 to 14.30), indicating inadequate oral health and care training despite having more experience in taking care of older people. Significant differences in practice were observed between the two groups after the OHE programme (*p* = 0.006). The three older residents exhibited poor oral health and multiple oral problems. Conclusions: This study revealed that the OHE programme effectively improved attitudes of the healthcare providers and provided a sustaining effect on attitude towards oral health and oral care. However, there were still inadequacies in oral hygiene provision by some healthcare providers, possibly due to unattended oral diseases and hygiene needs, as well as personal and environmental barriers that merit further investigation. Regular evaluation and enforcement of oral care/hygiene provision in LTCIs are necessary to maintain oral health and prevent dental and gum diseases in older residents. Immediate referral for dental treatment is recommended for older people with signs of dental/oral disease(s).

## 1. Introduction

The issue of oral health in the aging population has become a global concern [1,2,3]. Many older people aged 60 years or older suffer from multiple oral problems that require attention [4,5,6,7]. Common issues include oral discomfort or pain caused by dental caries, periodontal diseases, oral cancers, and oral infections [4,5,6,7,8]. Poor dental health is often due to inadequate oral hygiene habits and attitudes, such as neglecting to use dental floss or rinse the mouth properly while brushing [9]. Unfortunately, oral care is often overlooked, particularly in long-term care institutions (LTCIs) [10].

As research on oral health and its impact on general wellbeing has grown, it has become clear that oral health maintenance is essential in elderly care [5,6,7]. Many elderly residents in LTCIs are unable to perform adequate oral care due to physical, mental, or cognitive limitations, and therefore rely heavily on healthcare workers [8,9,10,11,12]. Unfortunately, oral care is often considered a low priority, leading to the development of oral diseases, physical pain, malnutrition, and esthetic problems [6,9,13]. This vicious cycle can make it difficult to achieve healthy aging [5]. The COVID-19 pandemic has only exacerbated the issue, with strict regulations and scarce manpower affecting the quality of oral care in LTCIs [14,15]. Therefore, it is crucial to re-examine and prioritize healthy-aging promotion in this special population group, even as the world begins to reopen. Healthcare workers in LTCIs play a crucial role in maintaining the oral health of residents, but inadequate knowledge and training can hinder their ability to provide proper oral care [9,13,14,15]. Poor knowledge, attitudes, and practice (KAP) have been reported among healthcare workers, highlighting the need for education programs to enhance their understanding of oral health and improve their attitudes and practice towards providing oral care to older residents [14,15,16,17]. Based on the interactions among these three constructs, KAP, education programmes are crucial to enhancing knowledge (about the basic concept of oral health, oral problems of older people, and oral care techniques) and substantially improving the attitudes and practice of healthcare workers in the oral care of older residents [18,19,20,21,22]. Although studies have been conducted on oral health educational programs, two systematic reviews on oral health interventions for healthcare staff in long-term care facilities have emphasized the importance of educational programs in maintaining the oral health of older residents. Furthermore, there is a lack of high-quality studies focusing on Asian older adults [23,24]. As the elderly population continues to grow, it is crucial to enhance healthcare services for oral health, including dental care utilization [23,25]. Additionally, there is a need to improve education and oral care services for older adults, especially those in long-term care facilities [26,27]. Therefore, the aims of this study were to evaluate the effects of an oral health education (OHE) programme on the KAP of healthcare workers in LTCIs and to assess the quality of oral care provided to residents by a small sample of healthcare workers after the OHE programme.

### The Oral Health Educational (OHE) Programme

The OHE programme was developed based on previously established protocols [19,20,21,22,28,29]. It consisted of two parts, namely education and oral care skill demonstration. The initial two sessions focused on education, where healthcare workers in the intervention group received fundamental knowledge on oral health, particularly related to common oral problems in older individuals. The following two sessions focused on skill demonstration, where healthcare workers were trained on performing oral care procedures for older residents. They then demonstrated their acquired skills, which were evaluated. Each session was conducted for one hour on a weekly basis. The specifics of the OHE programme can be found in Table 1. The teaching materials were designed in accordance with information provided by the Oral Health Education Division of the Department of Health [30]. The programme was delivered by the PI, who also served as the educator.

## 2. Materials and Methods

### 2.1. Study Design and Participants

The present study was a case-control study that conveniently sampled LTCIs to investigate the effects of an OHE programme on the KAP of healthcare workers regarding the oral health of LTCI residents, following the STROBE guidelines for case-control studies [31], provided in Supplementary S1. Two LTCIs were assigned to either the intervention or control group by a research assistant. Three partially dentate LTCI residents were conveniently recruited for supervised practical/training in the intervention group.

A total of 40 healthcare workers responsible for oral care for older residents were recruited, with 20 in the intervention and 20 in the control group. The intervention group received a four-lesson educational programme, while the control group received standard support. After the OHE programme, the chosen healthcare workers in the intervention group performed oral care on three older residents who were partly care-dependent and underwent oral examinations with photographic recordings. 

### 2.2. Instruments

The KAP survey utilized in this study was developed by Wong [32]. It comprises 19 knowledge-based items with response options of ‘Yes’, ‘Don’t know’, and ‘No’. Correct answers were scored as ‘1’, while incorrect or ‘Don’t know’ responses were scored as ‘0’. The attitudes and practice sections consisted of 13 and 19 items, respectively, with a 5-point Likert scale ranging from ‘1’ (strongly disagree) to ‘5’ (strongly agree). The KAP scale ranges were ‘0 to 19’, ‘13 to 65’, and ‘19 to 95’, respectively, with a total score range of ‘32 to 179’. The Cronbach’s alphas for knowledge, attitude, practice, and overall KAP were 0.67, 0.93, 0.92, and 0.94, respectively.

### 2.3. Study Procedure

This study was divided into two parts: (1) collecting surveys from healthcare workers, and (2) conducting oral examinations and standardized photographic recordings of the oral conditions of three conveniently selected older residents. Ethics approval was obtained from the research Ethics committee with reference number REC2021101. 

All eligible healthcare workers were required to complete the KAP survey on oral health and oral care of older residents at baseline and five weeks after the intervention group completed the OHE programme. Due to the COVID-19 pandemic, the last two lessons of the on-site OHE programme were postponed after the first two lessons of the skill demonstration. To maintain the sustainability of the OHE programme, video recordings of the first two lessons were made available to the healthcare workers at the intervention LTCI. When the social distancing regulation was relaxed, the last two lessons of the skill demonstration and return demonstration were conducted on site. A round-up session of the first two lessons was given to healthcare workers at the intervention LTCI to consolidate their knowledge before the third lesson of the oral care skill demonstration. The last lesson of the return skill demonstration for all healthcare workers at the intervention LTCI was conducted one week later.

After the OHE programme, two healthcare providers from the intervention group were invited to perform oral care for three older residents, who were partly self-care-dependent and were selected by the in-charge nurse. Informed consent (Supplementary S2) was obtained from the older residents and their families before the oral examination. Oral status was recorded with standardized clinical photography, with or without the application of a plaque-disclosing agent (Ci plaque checker, C.I. Medical Co. Ltd., Hong Kong, China), before and after oral care/hygiene delivery. The flow of the study is illustrated in Figure S1. To benefit the control LTCI care providers, the same four-session OHE programme was delivered before the conclusion of this study.

### 2.4. Statistical Analysis

The data analysis was conducted using SPSS v26.0 (IBM Corporation, Armonk, NY, USA). The normality of continuous variables was assessed using skewness statistics and normal Q-Q plots. No continuous variable was found to violate the normality assumption. Descriptive statistics were used to summarize the explanatory variables related to participants’ characteristics, such as age, gender, educational level, and working experience, and the outcome variables (KAP). The associations between the outcome variables (KAP) and demographic characteristics were assessed using Chi-square or univariate analyses, including Pearson’s correlation coefficient, independent-samples *t*-test, or one-way ANOVA, depending on the level of measurement of the outcome variables. All statistical tests were two-sided, and a *p*-value < 0.05 was considered statistically significant.

## 3. Results

### 3.1. Participants’ Demographic Characteristics (Table 2)

Forty participants from two medium-care-dependent LTCIs were recruited for the study. Of the 40 healthcare workers, 38 were females (95%). Although female healthcare workers were the majority (95%) in both groups, there was no significant difference between the genders in the entire sample or in the individual groups. The mean age of the participants was 54.7 ± 12.5 years (mean ± standard deviation [SD]). The demographic characteristics of the healthcare workers are presented in Table 2. Approximately 45% of healthcare workers in the intervention group had no prior experience working in LTCIs. More than 60% of healthcare workers in both groups had not received oral care training for older adults.

**Table 2:** Demographic and background of participants.

	Intervention Group (n = 20)		Control Group (n = 20)		*p*
	Count	%	Count	%	
**Gender**					1.00
Male	1	5.0	1	5.0	
Female	19	95.0	19	95.0	
**Age (year)**					0.187
≤55	6	30.0	8	40.0	
56–60	7	35.0	2	10.0	
61–65	3	15.0	7	35.0	
>65	4	20.0	3	15.0	
**Education level**					0.066
Primary	9	45.0	11	55.0	
Secondary	6	30.0	9	45.0	
Tertiary	5	25.0	0	0.0	
**Years of working in LTCI**					0.227
<2 years	5	25	9	45	
2-<5 years	2	10	1	5	
≥5 years	13	65	10	50	
**Experience of taking care of the elderly in LTCI**					<0.001 ***
No	15	75	3	15	
Yes	5	15	15	75	
**Oral care training**					0.520
No	14	70	12	60	
Yes	6	30	8	40	

LTCI: Long-term care institution; *** *p* < 0.001.

### 3.2. Differences of Knowledge, Attitudes, and Practice between the Two Groups and Pre- and Post-Intervention (Table 3)

Before the intervention, the mean KAP scores of the healthcare workers in the control group were higher than those in the intervention group. However, following the OHE programme, significant improvements were observed in the post-intervention mean scores of KAP for the intervention group. While significant differences in knowledge, practice, and overall KAP were observed between the two groups before the intervention, the only significant differences in practice were noted after the intervention. Table 3 presents the results of independent t-tests on KAP differences between the two groups. Knowledge (*p* < 0.001), practice (*p* < 0.001), and overall KAP (*p* < 0.001) scores were significantly higher in the control group pre-intervention, while practice (*p* = 0.006) remained significantly higher post-intervention in the control group. 

**Table 3:** KAP differences between the two groups and before and after intervention.

	Intervention Group (n = 20)		Control Group (n = 20)					
	Mean	SD	Mean	SD	*p*	95% CI	MD	t
**Pre-intervention**								
Knowledge (K)	13.55	4.51	17.25	2.15	<0.001 *	1.41 to 5.99	3.70	3.31
Attitudes (A)	47.10	4.78	49.30	4.85	0.026	−0.88 to 5.28	2.20	1.45
Practice (P)	42.85	5.35	48.4	5.62	<0.001 *	2.04 to 9.06	5.55	3.20
Overall KAP	103.50	12.54	114.95	8.50	<0.001 *	4.56 to 18.34	11.45	3.38
**Post-intervention**								
Knowledge	14.00	1.86	14.30	2.13	0.106	−0.98 to 1.58	0.30	0.47
Attitudes	51.0	4.22	51.35	4.32	0.133	−2.38 to 3.08	0.35	0.26
Practice	46.45	7.44	51.05	5.76	0.006 *	0.33 to 8.87	4.60	2.19
Overall KAP	111.45	10.68	116.70	7.55	0.014	−0.69 to 11.19	5.25	1.80
**Between two study periods (p)**								
Knowledge	0.119		<0.001 *					
Attitudes	0.002 *		0.030					
Practice	0.015		0.020					
Overall KAP	0.008 *		0.071					

* *p* was set at 0.05/6 or <0.0083; CI: Confidence interval; MD: Mean difference.

When comparing the two study periods using paired t-tests with a significance level of *p* < 0.0083, all participants (intervention + control, n = 40) showed significant improvements in attitudes (*p* = 0.001), practice (*p* = 0.003), and overall KAP (*p* = 0.005) scores, while no significant change was noted in knowledge scores (*p* = 0.018).

When comparing the two time periods within the same group using paired *t*-tests, significantly higher scores for attitudes (*p* = 0.002) and overall KAP (*p* = 0.008) were noted after intervention in the intervention group, while significantly lower scores in knowledge were observed for the control group (*p* < 0.001).

### 3.3. Oral Care Practice by Healthcare Workers

The three selected older residents (A, B, and C) were reported to have moderate levels of cognitive disability and were partly self-care-dependent, requiring assistance with personal hygiene and self-care activities. Oral care practices were evaluated through photographs taken after being performed by healthcare providers selected from the intervention group. Tables S1 and S2 provide detailed information on their oral conditions, based on oral examinations before and after the application of a plaque-disclosing agent. During the oral care procedures, the healthcare workers used only a toothbrush for cleaning and appeared hesitant, particularly when brushing the posterior teeth of the three older residents.

## 4. Discussion

This study demonstrated that the oral health educational programme improved the attitude and overall KAP of the healthcare workers in the intervention group, consistent with previous research [16,22]. However, there was no improvement observed in the knowledge and practice of the intervention group after the OHE programme, possibly due to the limitations of the COVID-19 pandemic that was indeed not supportive for effective knowledge acquisition as well as the practice of oral health delivery. The OHE programme should be simplified and improved to increase the awareness and interest of healthcare workers in oral health. Innovative modes of learning, such as virtual reality or simulation, could increase healthcare workers’ situational awareness and clinical skill translation in daily care. This learning mode provides more flexibility and applicability of the learning content in real practice [33]. Furthermore, healthcare workers implementing oral care in LTCI residents should receive training in appropriate oral care assessment and regular evaluation.

Interestingly, the control LTCI healthcare workers showed better knowledge and practice at baseline, and their practice remained significantly higher even after the intervention group received training (Table 3). However, their knowledge significantly deteriorated after the intervention, indicating that healthcare workers may lack confidence in their oral care knowledge without proper training. Personal beliefs and external barriers, such as fear of handling uncooperative older residents, can also affect the delivery of oral care practice [34,35,36]. In addition to personal beliefs, training and support of healthcare workers play a crucial role in the delivery of oral care practice to LTCI residents. Most healthcare workers are aged 40 years or older and typically receive oral care training in a brief period to quickly prepare them to work at LTCIs under the supervision of licensed nurses. Healthcare workers in LTCIs may have limited awareness or authority to improve the oral conditions of older residents and promote good oral health [8,18]. Their duties are based on tasks assigned by the nurse in charge, their daily routines, and the available manpower. The shortage of frontline healthcare workers has garnered significant attention from both professionals and the public since the COVID-19 pandemic. As such, ensuring good oral hygiene and care for older residents has become a new challenge. Caution must be exercised when interpreting the results of this study due to a small, convenient sample size.

The actual oral care practice performed by healthcare workers who received the OHE programme revealed some notable shortfalls. Firstly, during the procedure, older residents were seated without any support behind their heads, leading to increased fatigue, discomfort, and choking risk. This posture also made it difficult for healthcare workers to thoroughly assess the oral condition and clean the posterior part of the oral cavity. Healthcare workers may encounter various barriers that hinder their oral care practice [35,36]. Additionally, our observations showed that healthcare workers were hesitant to perform oral care in front of unfamiliar colleagues or afraid of accidentally hurting the older residents. For example, despite being educated to use dental floss or interdental brushes for better oral care, the healthcare workers only used toothbrushes and toothpaste, resulting in inadequate cleaning of the older residents’ oral cavity [37]. Therefore, improvements in healthcare workers’ confidence and training are crucial for better oral care practice.

The study highlights the poor oral conditions of partly self-care-independent older residents, who presented with multiple oral problems. This finding underscores the need for assistance in self-care, regular dental consultation, and potential nutritional concerns. The lack of regular assessment may be associated with the underdetermination of self-care dependence (such as BIAL) and cognitive status (such as MMSE) of the older residents, leading to inadequate self-care dependence for oral care [5,7,8]. Additionally, the instruction to open their mouths wide was not adhered to, limiting the effectiveness of brushing, particularly for the posterior teeth. Furthermore, routine oral health assessment was lacking, and various severities of periodontal and dental diseases were identified through photograph-based oral examination (Tables S1 and S2). Their oral conditions may impact on balanced diet/proper nutrition acquisition and requires follow-up accordingly [38]. These findings highlight the need for follow-up to ensure balanced diet and proper nutrition acquisition, using validated, objective tools for evaluating nutritional status and diet quality [39,40] and diet quality [41,42]. Lacking regular dental check-ups is an important reason for the poor oral health conditions of the three LTCI residents. Outreach Dental Care should be advocated to provide onsite dental health services with adequate support from the older residents and their families [43,44].

### Limitations

This study has provided significant insights into the KAP of healthcare workers and the oral health of older residents, which can increase awareness among the government and the general public. However, it is important to note that the findings are based on a small sample size from only two LTCIs, which limits the generalizability of the results to other healthcare professions and populations. Therefore, it is recommended to include a larger sample size in future studies to obtain more representative outcomes. Additionally, to gain a deeper understanding of the changes in healthcare workers’ KAP over time, a longitudinal study is highly recommended.

Before implementing any intervention, it is crucial to consider its cost-effectiveness. In this study, the effectiveness of the OHE programme was reported, as it is vital to enhance oral care competence. Based on the results, it is suggested that the OHE programme and its delivery mode can be modified and implemented in practice. However, it is important to estimate the costs according to institutional policies and practices before implementing the programme. 

## 5. Conclusions

The oral health of older individuals is a pressing issue in gerontological care. A recent study revealed that healthcare workers in long-term care institutions possessed reasonable KAP regarding oral health. However, the study also found that oral care practices for older residents still required greater enforcement. The oral conditions of these individuals were generally poor and potentially neglected due to ineffective care practices. Therefore, it is crucial to implement proper oral care practices, including comprehensive assessments and regular dental check-ups. To achieve this, healthcare workers should reinforce appropriate techniques of oral care practice, utilizing adequate equipment. Regular oral health assessments are essential to maintain care quality and facilitate continuous improvement. Policymakers must be made aware of these findings to improve current oral care practices using easily accessible and innovative methods to assist older individuals in need of proper oral care.

## Figures and Tables

**Table 1:** Details of the oral health education programme.

Sessions	Titles	Aims	Formats	Class Size	Duration
**One**	Basic knowledge in general concerning oral health of nursing home residents and the importance of oral care	To provide basic knowledge about importance of oral care in oral health of nursing home residents and equipment of oral care.	Face-to-face PowerPoint presentation with video recording for revision and self-learning *	Maximum 20	One hour
**Two**	Common oral problems among nursing home residents	To provide basic knowledge about common oral problems among nursing home residents and basic techniques of oral care to nursing home residents who may have different needs.	Face-to-face PowerPoint presentation with video recording for revision and self-learning *	Maximum 20	One hour
**Three**	Skills of oral care to nursing home residents who may have different conditions	To let healthcare providers learn skills and techniques of oral care for nursing home residents with different needs.	Face-to-face PowerPoint presentation	Maximum 10	One hour
**Four**	Return demonstration of oral care skills/techniques performed by LTCI healthcare workers	To evaluate oral care techniques and receive feedback for improvement	Face-to-face PowerPoint presentation with video recording for revision and self-learning	4–5	Flexible, until satisfaction of trainee/trainer

* Recorded video footages were used when condition not allowing extended face-to-face interactions or increased work demands at long-term care institution, e.g., during the height of COVID-19 pandemic.

## Data Availability

The data presented in this study are available on request from the corresponding author. The data are not publicly available due to the confidentiality reason of the participants.

## Supplementary Materials

The following supporting information can be downloaded at: https://www.mdpi.com/article/10.3390/geriatrics9010016/s1, Figure S1: The flow of the study; Table S1: Oral condition of three older residents based upon standardized clinical photographs before oral care/hygiene; Table S2: Oral condition of three older residents based upon standardized clinical photographs after oral care/hygiene by two healthcare providers from the intervention group after the training; Supplementary S1: STROBE-checklist-case-control study; Supplementary S2: Informed consents for healthcare workers and older residents.
