# Supplementary material for: Educational Programme on Knowledge, Attitudes, and Practice of Oral Care/Hygiene Provision by Healthcare Providers to Older Residents in Long-Term Care Institutions: A Case-Control Study
Source: Geriatrics (Basel). 2024 Jan 29;9(1):16. doi: 10.3390/geriatrics9010016 (PMC10888457; doi:10.3390/geriatrics9010016)
Supplement: Supplementary file 1 [file geriatrics-09-00016-s001.zip › geriatrics-2755858-supplementary.pdf]

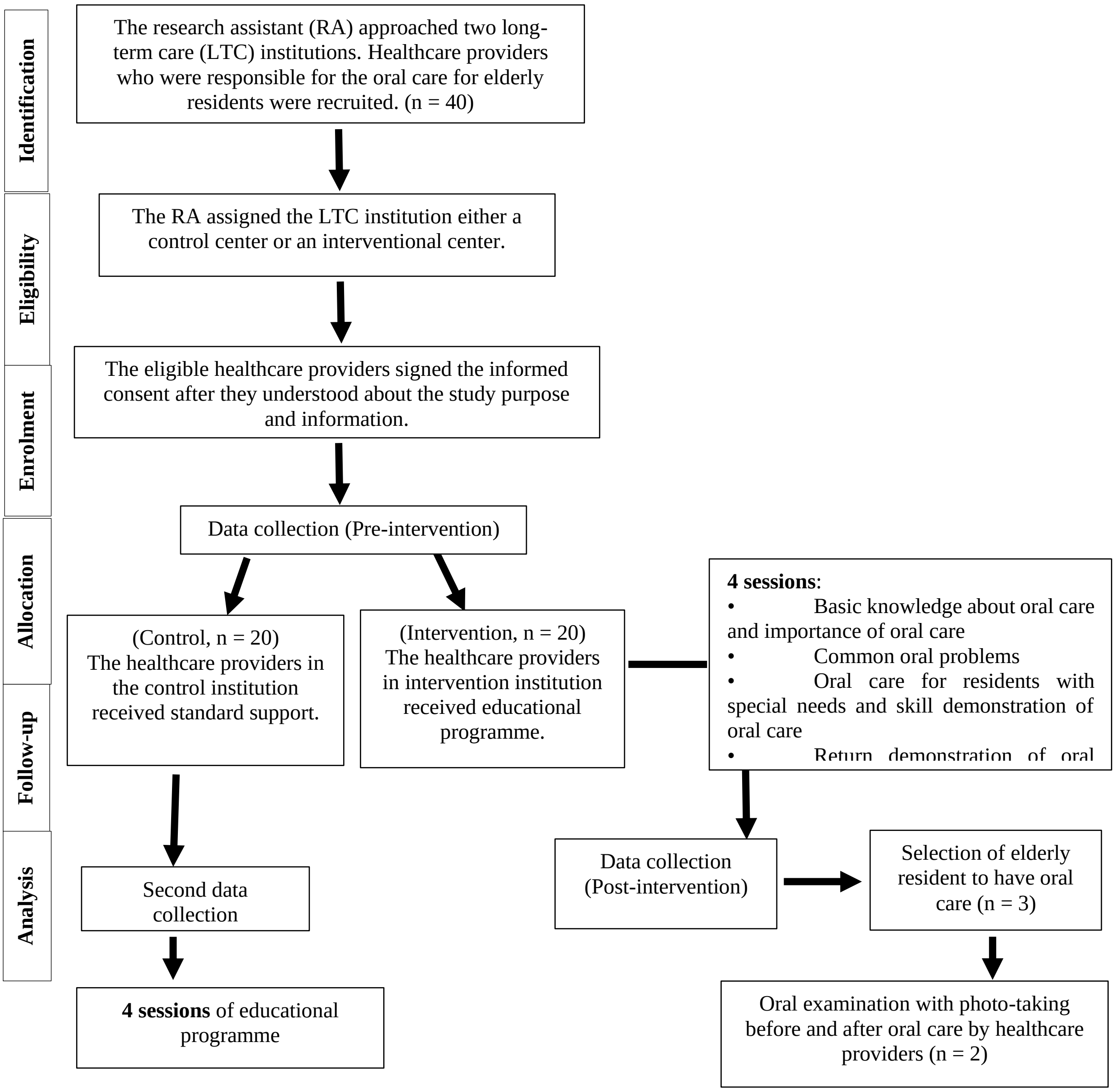

Figure S1. The flow of the study.

STROBE Statement—Checklist of items that should be included in reports of ***case-control studies***

	Item No	Recommendation	Page No.
**Title and abstract**	1	(*a*) Indicate the study's design with a commonly used term in the title or the abstract	1
		(*b*) Provide in the abstract an informative and balanced summary of what was done and what was found	1
**Introduction**			
Background/rationale	2	Explain the scientific background and rationale for the investigation being reported	1-2
Objectives	3	State specific objectives, including any prespecified hypotheses	3
**Methods**			
Study design	4	Present key elements of study design early in the paper	3
Setting	5	Describe the setting, locations, and relevant dates, including periods of recruitment, exposure, follow-up, and data collection	3
Participants	6	(*a*) Give the eligibility criteria, and the sources and methods of case ascertainment and control selection. Give the rationale for the choice of cases and controls	3
		(*b*) For matched studies, give matching criteria and the number of controls per case	3
Variables	7	Clearly define all outcomes, exposures, predictors, potential confounders, and effect modifiers. Give diagnostic criteria, if applicable	3
Data sources/ measurement	8*	For each variable of interest, give sources of data and details of methods of assessment (measurement). Describe comparability of assessment methods if there is more than one group	3
Bias	9	Describe any efforts to address potential sources of bias	3
Study size	10	Explain how the study size was arrived at	3
Quantitative variables	11	Explain how quantitative variables were handled in the analyses. If applicable, describe which groupings were chosen and why	4
Statistical methods	12	(*a*) Describe all statistical methods, including those used to control for confounding	4
		(*b*) Describe any methods used to examine subgroups and interactions	4
		(*c*) Explain how missing data were addressed	NA
		(*d*) If applicable, explain how matching of cases and controls was addressed	

		(*e*) Describe any sensitivity analyses	NA
**Results**			
Participants	13*	(a) Report numbers of individuals at each stage of study—eg numbers potentially eligible, examined for eligibility, confirmed eligible, included in the study, completing follow-up, and analysed	4
		(b) Give reasons for non-participation at each stage	NA
		(c) Consider use of a flow diagram	Figure S1
Descriptive data	14*	(a) Give characteristics of study participants (eg demographic, clinical, social) and information on exposures and potential confounders	
		(b) Indicate number of participants with missing data for each variable of interest	4 & Table 2
Outcome data	15*	Report numbers in each exposure category, or summary measures of exposure	4
Main results	16	(*a*) Give unadjusted estimates and, if applicable, confounder-adjusted estimates and their precision (eg, 95% confidence interval). Make clear which confounders were adjusted for and why they were included	4-5
		(*b*) Report category boundaries when continuous variables were categorized	4-5
		(*c*) If relevant, consider translating estimates of relative risk into absolute risk for a meaningful time period	

Other analyses	17	Report other analyses done—eg analyses of subgroups and interactions, and sensitivity analyses	5
**Discussion**			
Key results	18	Summarise key results with reference to study objectives	6-7
Limitations	19	Discuss limitations of the study, taking into account sources of potential bias or imprecision. Discuss both direction and magnitude of any potential bias	7
Interpretation	20	Give a cautious overall interpretation of results considering objectives, limitations, multiplicity of analyses, results from similar studies, and other relevant evidence	6-7
Generalisability	21	Discuss the generalisability (external validity) of the study results	7
**Other information**			
Funding	22	Give the source of funding and the role of the funders for the present study and, if applicable, for the original study on which the present article is based	8

*Give information separately for cases and controls.

Note: An Explanation and Elaboration article discusses each checklist item and gives methodological background and published examples of transparent reporting. The STROBE checklist is best used in conjunction with this article (freely available on the Web sites of PLoS Medicine at http://www.plosmedicine.org/, Annals of Internal Medicine at http://www.annals.org/, and Epidemiology at http://www.epidem.com/). Information on the STROBE Initiative is available at http://www.strobe-statement.org.

Supplementary S2

TUNG WAH COLLEGE

Information Sheet and consent form (healthcare workers)

Educational programme on knowledge, attitude, and practice of healthcare workers in oral care of older residents in nursing homes

You are invited to participate in a research study conducted by Dr. Florence Wong, Assistant Professor of School of Nursing at Tung Wah College (TWC). Your participation is entirely voluntary. Before you decide, it is important that you understand why the research is being done and what it would involve for you. Please take time to read this information, and contact by Dr. Florence Wong at TWC, (address: Room 1605, School of Nursing, Tower 2, Kowloon Commerce Centre, 51 Kwai Cheong Road, Kwai Chung, Hong Kong, telephone number: 34686838, and email: florencewong@twc.edu.hk) if you have any enquiries.

PURPOSE OF THE STUDY

This study aims to examine the validity and viability of a newly developed educational programme on knowledge, attitude, and practice (KAP) of healthcare providers in oral care for older residents in long-term care (LTC) institutions.

PROCEDURES

You will receive the study information for your involvement and the study purpose. Once you agree to join in this study, you will be required to fill in one set of questionnaires including a demographic form and a KAP questionnaire. It takes about 30 minutes to finish. Then, you will receive 4-lesson educational programme related to oral care and will be required to fill in the KAP questionnaire in different time periods (3 and 6 months after the educational programme). The educational programme consists of two parts, education of oral care and oral care skill.

POTENTIAL RISKS / DISCOMFORTS AND THEIR MINIMIZATION

There is no foreseeable risk of harm or discomfort.

In case any bad effects of the procedure, you can seek help from Dr. Florence Wong and your participation will be immediately terminated to protect you from further risk of harm.

COMPENSATION FOR PARTICIPATION

Your participation is free of charge and is voluntary. There is no compensation for your involvement.

POTENTIAL BENEFITS

There is no potential benefit from your participation. This study can help test the validity and viability of the educational programme on KAP of healthcare workers toward oral care of older residents in long-term care institutions in the future studies.

CONFIDENTIALITY

All eligible healthcare workers will be assured of anonymity and confidentiality and implied consent will be used before data collection. All obtained personal information in the study will be used for research purposes only and strictly confidential. Subjects' name and contact numbers will be needed to follow up the data after the pilot intervention and oral care evaluation. Coding system will be used to identify the subjects for data entry also. All data will be accessed by researchers only. Once the data collection and data entry are completed, all data with healthcare workers' names and contact numbers will be permanently destroyed.

Responsible members of TWC may be given access to data for monitoring and/or audit of the study to ensure that the research is complying with applicable regulations.

DATA RETENTION AND STORAGE

All data will be stored in a locked cabinet. Soft copies of the research documents and electronic data files will be encrypted and stored in a password protected device. All data will be destroyed permanently after seven years. The signed informed consent forms will be discarded after 10 years. Personal identifiers will be removed upon the completion of study.

PARTICIPATION AND WITHDRAWAL

Your participation is voluntary. This means that you can choose to stop at any time without negative consequences. Note that if you anonymise during the study, it will not be possible to withdraw your data.

If you decide to participate, you may withdraw from the study at any time without penalty and without loss of benefits to which you are otherwise entitled.

FUNDING SOURCE

This study is supported by the College Research Grant (CRG).

QUESTIONS AND CONCERNS

This study has been reviewed and approved by Research Ethics Committee (REC), TWC, to protect participants' interests.

If you have any questions or concerns about the research, please feel free to contact Principal Investigator, Dr. Florence Wong at TWC, (address: Room 1605, School of Nursing, Tower 2, Kowloon Commerce Centre, 51 Kwai Cheong Road, Kwai Chung, Hong Kong, telephone number: 34686838, and email: florencewong@twc.edu.hk). If you have questions about your rights as a research subject, contact the Secretary, Research Ethics Committee (REC), TWC (ro@twc.edu.hk).

SIGNATURE

I ___________________________________ (Name of Subject) understand the procedures described above and agree to participate in this study. I ** wish / do not wish to be identified. (if the procedure will involve personal verification)

(** Please delete as appropriate.)

Signature: ________________

Date: ______________________

TUNG WAH COLLEGE

Information sheet and consent form (older residents)

Educational programme on knowledge, attitude, and practice of healthcare workers in oral care of older residents in nursing homes

You are invited to participate in a research study conducted by Dr. Florence Wong, Assistant Professor of School of Nursing at Tung Wah College (TWC). Your participation is entirely voluntary. Before you decide, it is important that you understand why the research is being done and what it would involve for you. Please take time to read this information, and contact by Dr. Florence Wong at TWC, (address: Room 1605, School of Nursing, Tower 2, Kowloon Commerce Centre, 51 Kwai Cheong Road, Kwai Chung, Hong Kong, telephone number: 34686838, and email: florencewong@twc.edu.hk) if you have any enquiries.

PURPOSE OF THE STUDY

This study aims to examine the validity and viability of a newly developed educational programme on knowledge, attitude, and practice (KAP) of healthcare providers in oral care for older residents in long-term care (LTC) institutions.

PROCEDURES

You will receive the study information for your involvement and the study purpose. Once you agree to join in this study, you will be participated in the oral care performed by a healthcare provider. The gum red cote disclosing agents will be used before the oral care. Oral photos will be taken before and after the oral care. The photos will be examined by an expert from the Dentistry at the University of Hong Kong. The duration of the whole procedure will be about 30-40 minutes.

POTENTIAL RISKS / DISCOMFORTS AND THEIR MINIMIZATION

There is no foreseeable risk of harm or discomfort.

In case any bad effects of the procedure, you can seek help from Dr. Florence Wong and your participation will be immediately terminated to protect you from further risk of harm.

COMPENSATION FOR PARTICIPATION

Your participation is free of charge and is voluntary. There is no compensation for your involvement.

POTENTIAL BENEFITS

There is no potential benefit from your participation. This study can help test the feasibility and practicability. The results help understand more about KAP of caregivers toward oral care of older residents in long-term care institutions in the future studies.

CONFIDENTIALITY

All subjects will be assured of anonymity and confidentiality and implied consent will be used before data collection. All obtained personal information in the study will be used for research purposes only and strictly confidential. Coding system (A001, A002, A003, …) will be used to identify the subjects for data entry and verify the same subjects before and after oral care. All data will be accessed by researchers only. All data will be stored in the PI’s office and will be permanently destroyed after seven years.

Responsible members of TWC may be given access to data for monitoring and/or audit of the study to ensure that the research is complying with applicable regulations.

DATA RETENTION AND STORAGE

All data will be stored in a locked cabinet. Soft copies of the research documents and electronic data files will be encrypted and stored in a password protected device. All data will be destroyed permanently after seven years. The signed informed consent forms will be discarded after 10 years. Personal identifiers will be removed upon the completion of study.

PARTICIPATION AND WITHDRAWAL

Your participation is voluntary. This means that you can choose to stop at any time without negative consequences. Note that if you anonymise during the study, it will not be possible to withdraw your data.

If you decide to participate, you may withdraw from the study at any time without penalty and without loss of benefits to which you are otherwise entitled.

PARTICIPATION AND WITHDRAWAL

Your participation is voluntary. You are voluntary to participate in a session of oral care and oral examination. You have the right to withdraw from the study without penalty of any kind.

QUESTIONS AND CONCERNS

This study has been reviewed and approved by Research Ethics Committee (REC), TWC, to protect participants' interests.

If you have any questions or concerns about the research, please feel free to contact Principal Investigator, Dr. Florence Wong at TWC, (address: Room 1605, School of Nursing, Tower 2, Kowloon Commerce Centre, 51 Kwai Cheong Road, Kwai Chung, Hong Kong, telephone number: 34686838, and email: florencewong@twc.edu.hk). If you have questions about your rights as a research subject, contact the Secretary, Research Ethics Committee (REC), TWC (ro@twc.edu.hk).

SIGNATURE

I ___________________________________ (Name of Subject) understand the procedures described above and agree to participate in this study.

I ** agree / do not agree to participate in this study with oral care and oral phototaking. I understand the purpose and nature of the study from information sheet and my participation is voluntary indicating that I am free to withdraw at any time.

(** Please delete as appropriate.)

Signature: _______________

Date: ___________________

Table S1. Oral condition of three older residents based upon standardized clinical photographs before oral care/hygiene.

**Before oral care/hygiene**		
**Older residents**	**Description of the oral condition**	**Impression**
**A**<br>An 86 years old man who has multiple systemic diseases, including dementia with partly self-care dependence.<br><br>He had been living at the LTCI for 1 year<br>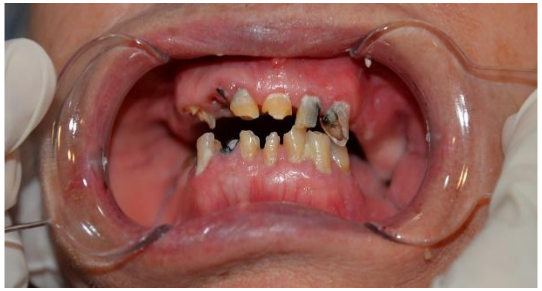	• Poor overall oral cleanliness and health;<br>• Generalized visible supragingival plaque.;<br>• Partially dentated;<br>• All remaining teeth either severely attrited/broken or grossly carious teeth with multiple retained roots;<br>• Collapsed bite due to missing/attrited/carious cheek teeth<br>• A lesion compatible to a draining/discharging sinus observable on the gum, at lip/labial side, close to the anticipated root apex of the left maxillary central incisor;<br>• Generalized red swollen gum compatible to significant gingival and/or periodontal inflammation/infection especially between lower left lateral incisor and canine, lower left canine and first premolar, distal side of right upper maxillary central incisor, to name a few.	Uncontrolled dental caries, given rise to many retained roots, and at least one draining endodontic infection which provide local foci of infection. The latter could also be caused by periodontal infection due to poor gum health. Long-term unattended dental/oral conditions gave rise to over closure of the mouth.<br><br>Lack of dental attention, prevention and rehabilitation lead to severe wear of the remaining teeth which could not fulfil daily eating/chewing demands.<br><br>Older resident A is at high risks of dental pain and discomfort, and perhaps dietary restrictions too due to poor masticatory efficiency.
**B**<br>A 75- years old lady who has hypertension, schizophrenia and is partly self-care dependence.<br><br>She had been living at the LTCI for 5 years.	• Poor overall oral cleanliness and health.<br>• Generalized visible supragingival plaque.;<br>• Partially dentated;<br>• Generalized severe gum inflammation.<br>• Upper and lower: first molar to first molar dentition, but with four retained roots with at least 2 with draining/discharging sinus, 12 standing teeth with caries;	Poor periodontal health, uncontrolled dental caries with both causing local chronic infections. Lack of dental attention and prevention.<br><br>Older resident B is at high risks of dental pain, discomfort and infection that could affect his/her diet as well as general wellbeing.

<table>
<tr>
<td>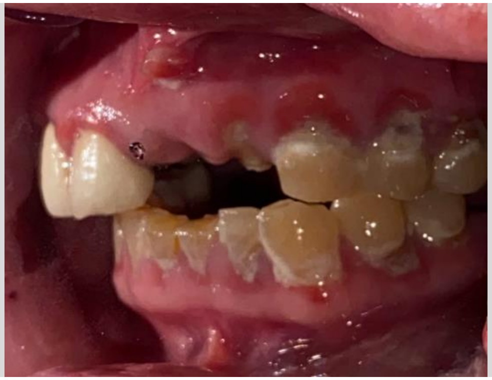</td>
<td>• Crowns on upper right canine, lateral and central incisor (joined)
• Attrition on lower left central incisor to lower right second incisor</td>
<td></td>
</tr>
<tr>
<td>C
A 77-year-old lady who had dementia with partly self-care dependence.

She had been living at the LTCI for 2.5 years.

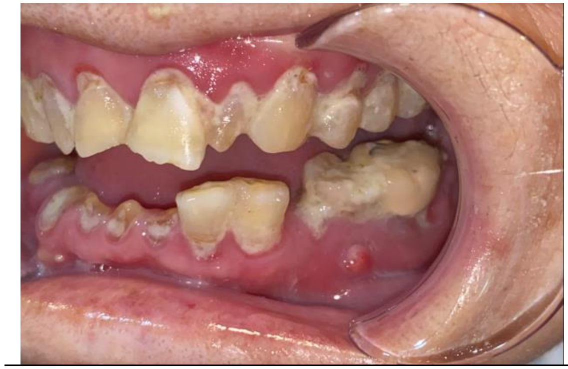</td>
<td>• Very poor overall oral cleanliness and health.
• Generalized visible supragingival plaque.;
• Partially dentated;
• Generalized moderate-severe gum inflammation.
• Upper: first molar to first molar dentition, lower: first left molar to second right premolar dentition, but with 7 of them retained roots at the lower arch with draining/discharging sinus at lower left second premolar, 3 standing teeth with caries;
• Crowns on lower left first molar.
• Attrition on both upper central incisors</td>
<td>Uncontrolled dental caries, given rise to many retained roots, and at least one draining endodontic infection which provide local foci of infection. The latter could also be caused by periodontal infection due to poor gum health.

Lack of dental attention, and prevention lead to caries and retained roots especially in lower right causing problems in eating/chewing.

Older resident C is at high risks of dental pain and discomfort, and perhaps dietary restrictions too due to poor masticatory efficiency.</td>
</tr>
</table>

Table S2. Oral condition of three older residents based upon standardized clinical photographs after oral care/hygiene by *two healthcare providers from the intervention group after the training.

After oral care/hygiene		
**Older residents**	**Oral examination**	**Description of oral condition**
**A**	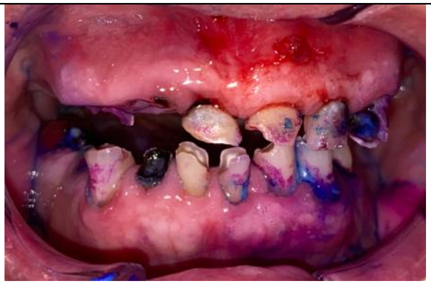	The healthcare provider, M, attempted to clean this client's mouth with twice mouth rinsing and completed the procedure in 3 minutes. She used the toothbrushes and toothpaste to clean the mouth after disclosing agent was applied. The healthcare provider M appeared more confident and asked the client to open his mouth for oral care. However, the oral health assessment had not been performed. It was difficult for them to understand oral condition of the client and prepare more suitable equipment for the procedure. In the oral care procedure, the healthcare worker A showed a little of embarrassment.<br><br>• Overall good amount of supra-gingival plaque was removed;<br>• Significant amount of plaque on decayed/carious lesions which may need professional attention; the similar could be observed around broken-down teeth which post difficulties for the LTCI healthcare providers to tackle;<br>• The similar could be observed when there is marked gum inflammation, quite a significant among of plaque could remained not able to be cleared away.
**B**	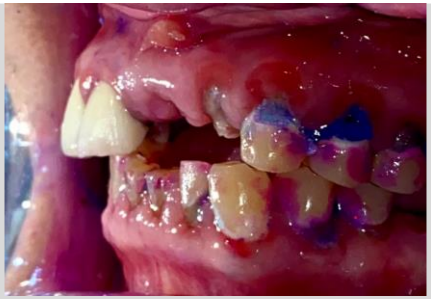	Again, the healthcare provider, M, attempted to clean this client's mouth with twice mouth rinsing and completed the procedure in 2 minutes. She used the toothbrushes and toothpaste to clean the mouth after disclosing agent was applied. The healthcare provider M was able to ask the client to open his mouth for oral care. However, the oral health assessment had not been performed. It was difficult for them to understand oral condition of the client and prepare more suitable equipment for the procedure. In the oral care procedure, the healthcare worker A showed a little of embarrassment.<br><br>• Overall good amount of supra-gingival plaque was removed;<br>• Significant amount of plaque on decayed/carious lesions which may need professional attention; the similar could be observed around broken-down teeth which post difficulties for

		the LTCI healthcare providers to tackle; the crowned teeth, as expected, due to the nature of the ceramic material, is relatively easy to be cleansed;<br>● The similar could be observed when there is marked gum inflammation, quite a significant among of plaque could remained not able to be cleared away.
**C**	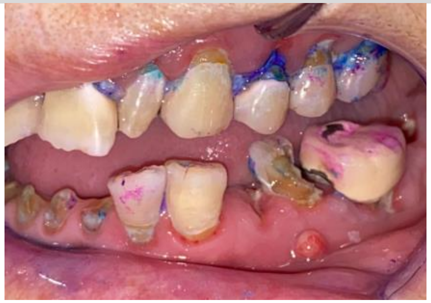	The healthcare provider, N, attempted to clean this client's mouth with thrice oral rinsing and completed the procedure in 3 minutes. She used the toothbrushes and toothpaste to clean the mouth after disclosing agent was applied. The healthcare provider N appeared inadequate confident for oral care. However, the oral health assessment had not been performed. It was difficult for them to understand oral condition of the client and prepare more suitable equipment for the procedure. In the oral care procedure, the healthcare worker N showed quite embarrassed.<br><br>● Overall good amount of supra-gingival plaque was removed;<br>● Significant amount of plaque on decayed/carious lesions which may need professional attention; the similar could be observed around broken-down teeth which post difficulties for the LTCI healthcare providers to tackle; the crowned teeth, as expected, due to the nature of the ceramic material, is relatively easy to be cleansed except the worn areas;<br>● The similar could be observed when there is marked gum inflammation, quite a significant among of plaque could remained not able to be cleared away.

*Two healthcare workers from the intervention group were selected to perform oral care for the three elderly residents. One of the healthcare worker performed oral care to the elderly residents A & B; the other healthcare workers performed oral care to elderly resident C.
